# Post-traumatic distal radioulnar synostosis in a child: a rare case report and literature review

**DOI:** 10.1093/omcr/omaf241

**Published:** 2025-11-26

**Authors:** Mouna Lazrak, Hidaya Zitan, Sarah Hosni, Karima Benali, Nidale Mrani Alaoui, Marouan Nour, Mohammed Anouar Dendane, Tarik Madhi, Abdelouahed Amrani

**Affiliations:** Faculté de Medecine et de Pharmacie de Rabat, Université Mohammed V de Rabat. Av. Hafiane Cherkaoui, Rabat 10000, Morocco; Pediatric Surgery, Ibn Sina University Hospital Center, BP 6527, Rue Lamfadel C herkaoui Rabat Institut, Rabat, Rabat 1005, Morocco; Faculté de Medecine et de Pharmacie de Rabat, Université Mohammed V de Rabat. Av. Hafiane Cherkaoui, Rabat 10000, Morocco; Pediatric Surgery, Ibn Sina University Hospital Center, BP 6527, Rue Lamfadel C herkaoui Rabat Institut, Rabat, Rabat 1005, Morocco; Faculté de Medecine et de Pharmacie de Rabat, Université Mohammed V de Rabat. Av. Hafiane Cherkaoui, Rabat 10000, Morocco; Pediatric Surgery, Ibn Sina University Hospital Center, BP 6527, Rue Lamfadel C herkaoui Rabat Institut, Rabat, Rabat 1005, Morocco; Faculté de Medecine et de Pharmacie de Rabat, Université Mohammed V de Rabat. Av. Hafiane Cherkaoui, Rabat 10000, Morocco; Pediatric Surgery, Ibn Sina University Hospital Center, BP 6527, Rue Lamfadel C herkaoui Rabat Institut, Rabat, Rabat 1005, Morocco; Faculté de Medecine et de Pharmacie de Rabat, Université Mohammed V de Rabat. Av. Hafiane Cherkaoui, Rabat 10000, Morocco; Pediatric Surgery, Ibn Sina University Hospital Center, BP 6527, Rue Lamfadel C herkaoui Rabat Institut, Rabat, Rabat 1005, Morocco; Faculté de Medecine et de Pharmacie de Rabat, Université Mohammed V de Rabat. Av. Hafiane Cherkaoui, Rabat 10000, Morocco; Pediatric Surgery, Ibn Sina University Hospital Center, BP 6527, Rue Lamfadel C herkaoui Rabat Institut, Rabat, Rabat 1005, Morocco; Faculté de Medecine et de Pharmacie de Rabat, Université Mohammed V de Rabat. Av. Hafiane Cherkaoui, Rabat 10000, Morocco; Pediatric Surgery, Ibn Sina University Hospital Center, BP 6527, Rue Lamfadel C herkaoui Rabat Institut, Rabat, Rabat 1005, Morocco; Faculté de Medecine et de Pharmacie de Rabat, Université Mohammed V de Rabat. Av. Hafiane Cherkaoui, Rabat 10000, Morocco; Pediatric Surgery, Ibn Sina University Hospital Center, BP 6527, Rue Lamfadel C herkaoui Rabat Institut, Rabat, Rabat 1005, Morocco; Faculté de Medecine et de Pharmacie de Rabat, Université Mohammed V de Rabat. Av. Hafiane Cherkaoui, Rabat 10000, Morocco; Pediatric Surgery, Ibn Sina University Hospital Center, BP 6527, Rue Lamfadel C herkaoui Rabat Institut, Rabat, Rabat 1005, Morocco

**Keywords:** radioulnar synostosis, pediatric trauma, distal forearm, surgical resection, interposition graft, forearm rotation

## Abstract

Radioulnar synostosis is a rare but severe complication of pediatric forearm trauma that results in the loss of forearm rotation and functional impairment. We report the case of a 7-year-old boy who developed post-traumatic distal radioulnar synostosis following a high-energy road traffic accident. Initial management involved open reduction and internal fixation of both forearm bones. One year later, due to loss of pronation-supination, the child underwent revision surgery with bony bridge resection and interposition. Sixteen months after the second surgery, the outcome was excellent with full restoration of forearm rotation and no recurrence. This case highlights the diagnostic and therapeutic challenges in pediatric synostosis and supports the role of early surgical resection with interposition for optimal results.

## Introduction

Radioulnar synostosis is a rare complication of forearm trauma in children, defined by abnormal osseous or fibrous fusion between the radius and ulna, leading to restricted pronation and supination. It may be congenital, iatrogenic, or post-traumatic. Post-traumatic synostosis is typically associated with high-energy injuries and carries significant functional implications. Risk factors include Monteggia-type injuries, comminuted fractures, soft tissue trauma, and improper surgical management. Despite being rare, pediatric cases require timely diagnosis and tailored treatment, considering growth potential and risk of recurrence.

## Case presentation

**Figure 1:**
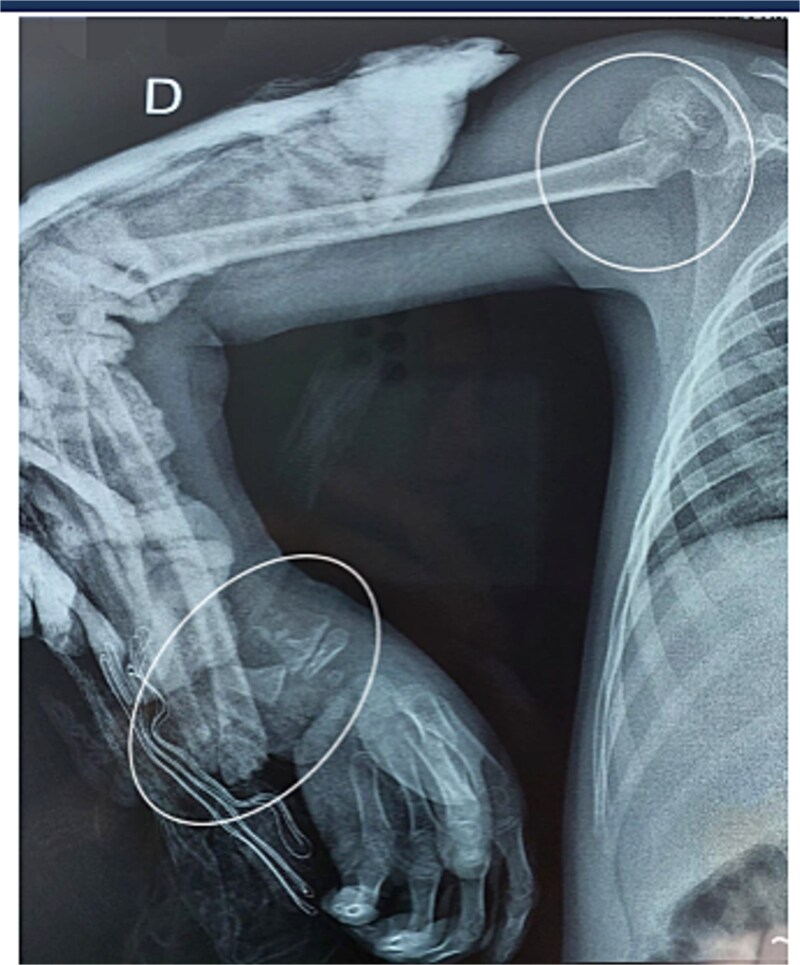
Initial radiograph showing proximal humerus fracture and distal radius and ulna fractures (‘floating elbow’).

**Figure 2:**
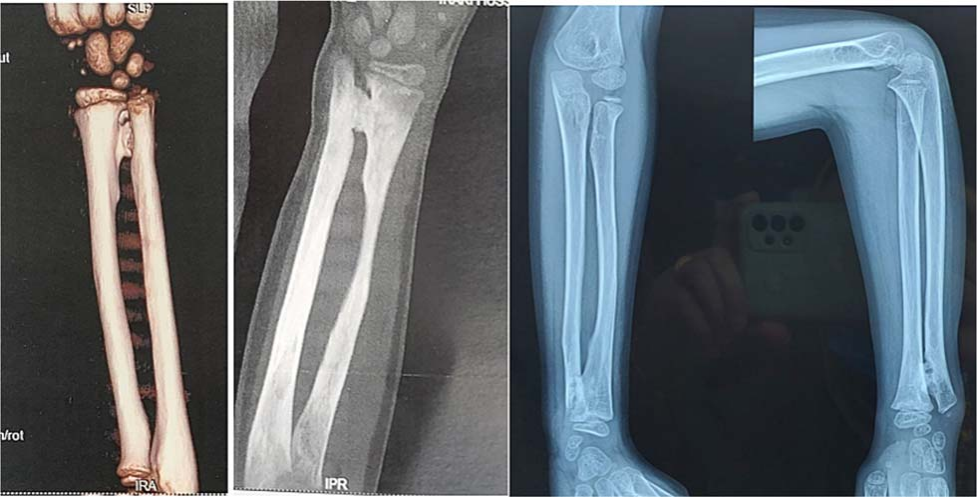
CT and X-ray images demonstrating distal radioulnar synostosis.

**Figure 3:**
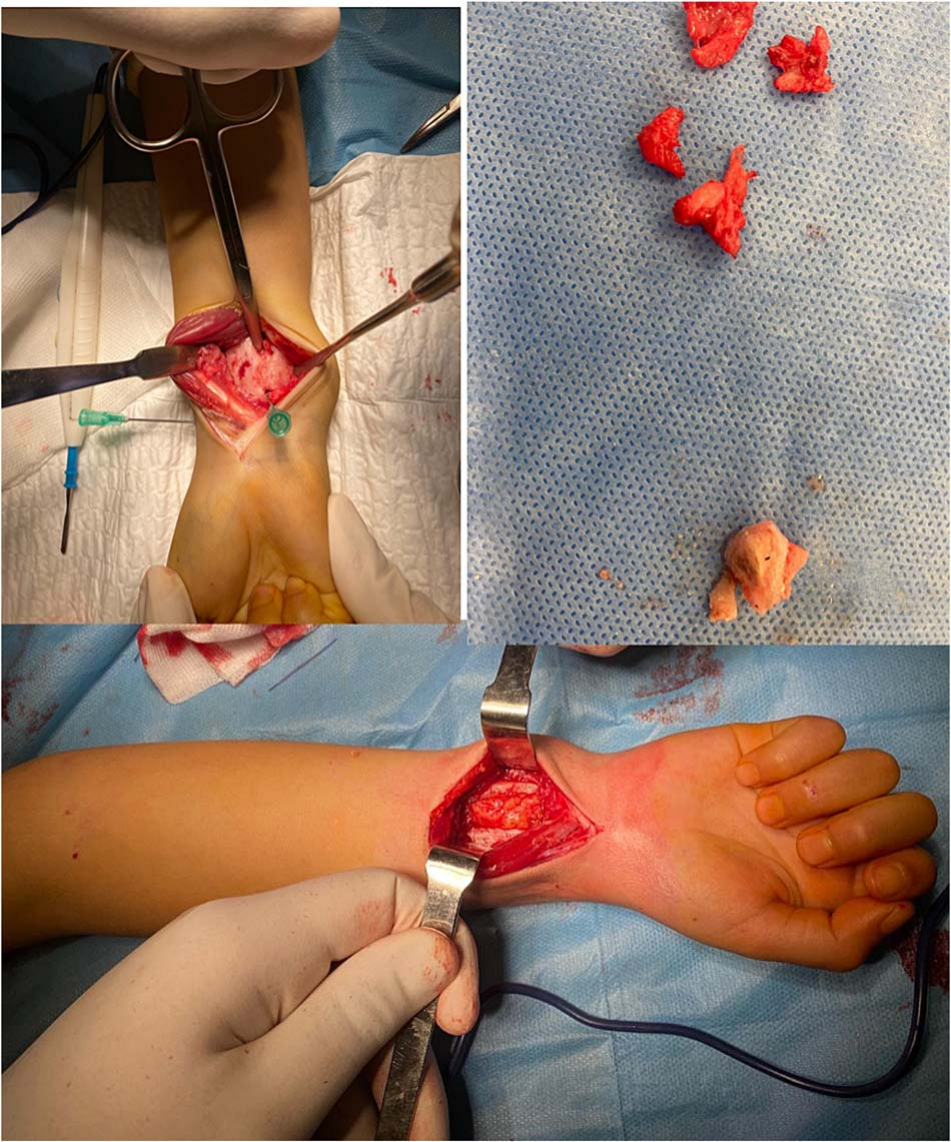
Intraoperative view of synostosis resection.

**Figure 4:**
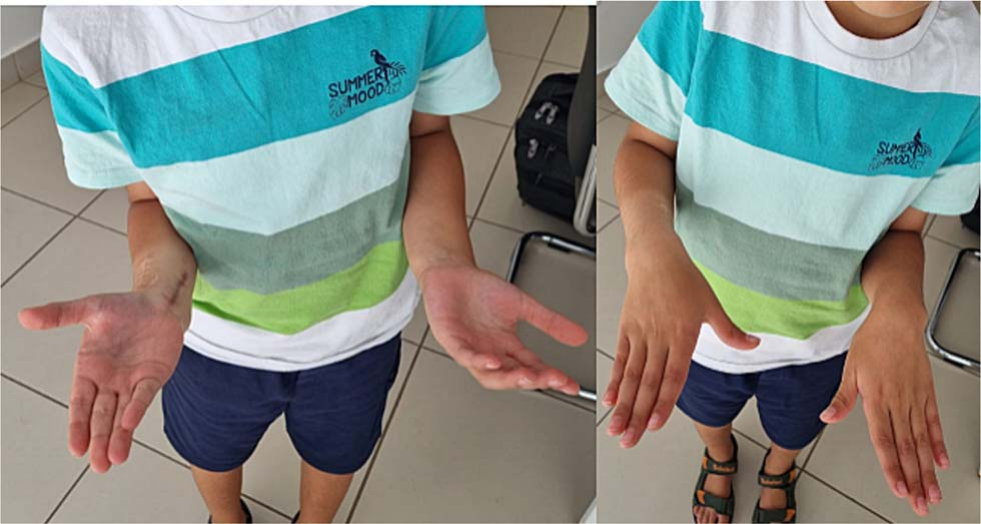
Postoperative image demonstrating restored pronation-supination.

A 7-year-old right-handed boy was involved in a high-energy road traffic accident, sustaining a fracture of the proximal humerus and an open fracture of the distal radius and ulna. This resulted in an atypical ‘floating elbow’ pattern. Initial radiographs showed proximal humerus fracture and distal radius and ulna fractures (‘floating elbow’) (Fig. 1). The initial treatment included open reduction and intramedullary pinning of both forearm bones. During follow-up, the patient presented with restricted pronation and supination. Imaging revealed a bony bridge at the distal radioulnar junction. CT and X-ray images demonstrated distal radioulnar synostosis (Fig. 2). A revision procedure was performed one year later, involving resection of the synostosis and interposition of soft tissue. An intraoperative view of the synostosis resection is shown in (Fig. 3). Postoperative recovery was uneventful, and at 16 months, the patient regained full forearm rotation with no evidence of recurrence. Postoperative images demonstrated restored pronation-supination (Fig. 4).

**Figure 5:**
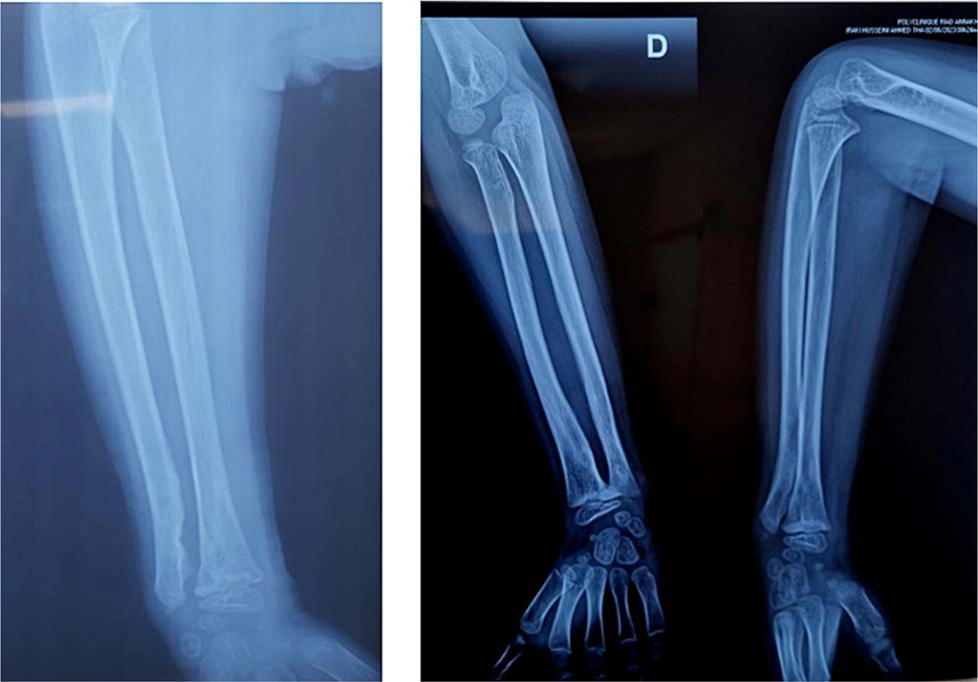
Radiographic follow-up 16 months after surgery showing no recurrence.

Radiographic follow-up 16 months after surgery showed no recurrence (Fig. 5).

**Table:** 

Movement	Preoperative ROM	Postoperative ROM
Pronation	0°	85°
Supination	0°	90°
Elbow Flexion	135°	135°
Elbow Extension	0°	0°

## Discussion

Post-traumatic radioulnar synostosis is an uncommon but functionally disabling complication of pediatric forearm fractures. It typically results from high-energy trauma, complex fracture patterns, and inadequate early management [1]. Although more frequently described in adults, pediatric cases represent a distinct challenge due to bone growth, remodeling potential, and long-term functional impact [2].

In our case, the synostosis developed at the distal radioulnar joint, which is particularly rare. Most reported pediatric cases involve proximal or mid-shaft locations [3]. The condition manifests clinically as a progressive limitation of pronation-supination, often painless, as seen in our patient. Imaging, especially CT, is crucial to confirm the diagnosis and evaluate the extent of bony bridging [4].

**Table 1:** summarizes recent pediatric cases of post-traumatic radioulnar synostosis for comparison.

Study (Year)	Age	Location	Treatment	Interposition Material	Outcome
Gounot et al. (2022)	6–8	Distal forearm	Resection	None	Good ROM
Dharmshaktu et al. (2024)	11	Mid-distal forearm	Resection + tendon graft + fat	Palmaris longus + fat	Full ROM at 10 yrs
Kell et al. (2024)	9	Proximal forearm	Resection	Not reported	Improved ROM
Current case (2025)	7	Distal forearm	Resection + fat graft	Autologous fat	Full ROM at 16 mo

The literature suggests an incidence of 3–9% for post-traumatic radioulnar synostosis following forearm fractures, particularly in cases with Monteggia injuries or open fractures with soft tissue trauma [1–3]. Several pediatric case reports and small series have been published in recent years. Gounot et al. (2022) described two cases of distal synostosis treated with simple resection and early rehabilitation, with satisfactory results. Dharmshaktu et al. (2024) reported a case treated with resection and interposition of palmaris longus tendon and free fat graft, achieving full range of motion at 10 years. Kell et al. (2024) highlighted early surgical planning in a series of pediatric patients, including one with a distal location. Recent pediatric cases of post-traumatic radioulnar synostosis are summarized in Table 1.

Surgical resection remains the gold standard treatment. However, recurrence is a known complication. Interposition of biological material (fat, fascia, muscle flaps) or synthetic barriers (silicone, polyethylene) is recommended to reduce re-ossification risk [2, 4]. In our patient, we used autologous subcutaneous fat, a simple and effective option with low morbidity. Functional recovery was excellent, with restoration of full rotation and no recurrence at 16 months.

Early and tailored rehabilitation is crucial, starting within days after surgery, with progressive mobilization. Follow-up during growth is essential to monitor for late recurrence or growth disturbance.

## Conclusion

Post-traumatic distal radioulnar synostosis in children is an uncommon but potentially debilitating condition. Timely diagnosis, appropriate surgical resection with interposition, and dedicated rehabilitation are critical for restoring function and preventing recurrence. This case adds to the limited pediatric literature and supports early surgical intervention in selected cases with good outcomes.

## Data Availability

Not applicable.

## Authors’ information

All authors are pediatric surgery specialist at CHU Ibn Sina, Rabat.
